# Record of Meteorological Conditions at Buffalo, for Nov., 1855

**Published:** 1856-01

**Authors:** Sanford B. Hunt


					﻿ART. IX. — Record of Meteorological Conditions at Buffalo, for Nov.,
1855. By Sanford B. Hunt, M. D.
(	* p-h ® EZ. ■ — -Pc s ? ® 2t ?
= 5	£ g a= 2’&£	x ®	®	~
O w Z CqOo — ox'*- 4J	c: x	~	cS
de ®~ Z-£2~ ?	= 2~ ? = t-g 5- c = © ®
S>®—-fec^i® og2'-t*‘ccZ°© — 2-0-
*> ’&A -	: 'SS2 “ = j.'p
S-Js’-g g.® “ ef-5 i* g^s ® S^s	§
2	’■= g^2	®2 2^	“-=2 BP23 2 “J S3-
«	S «, " £	“2 2 ^ £ * gjHHJh'2 § 22
S	~ = S"°c£’Sf=&acd3Et®®£'rC-§g§’®1?
,2 5 O jj S'- gjrt — £ fe ra — 5 £>£ ® cs fees ~ —
^Sp-^pohgSu— P"sSrfe P-.® . ® 2 o
:-2	®	®	£^	|	>>=	*	„•	^c-2£
q — ®	-;	® ® .£	—	-	-	fe:	—	b£— x	®	5 ’fe	2	"	-^	g i£~ 3
— —	Bp< p	>	a	2	p	c^ps.® 2	®	tn	c	* = c a
O"	cs	— !> "	tf	u	72	©	P_~	R o	—	~ r®	nz	.2	.S	®.5 tn R
•ajnjBjjduiaj, I	|S
jo oSubj jsejKoir)]	© ■^mt^coao —	CT — m©	© o — t— m	co mao	© ©	m —
. ’UOJJB.dBAJJ JOI	CO CO CO t-^	I—	t— L— CO	CO	t— CO _ CO	00
5 dui<L ubsj\[ ci ood--2cd’C©CT©'-di© ci — cd cd oo —	© <-' co'ci cc £	©
-	I co -r o to t-. f -? t -r to o -* -of1 co co CT co coco co co (M co -y
<
®	-oanjB i n	t-~t~- t— tr t—1~- co co co t-1- co t- t- _	t—_ . co co ©
-•lading^ ucajH	—■'	CT cd cd-d cd ct'-d-d'-d ©	©	tri td ci — CT	—’ ©	ad —	ci cd	co
i ______________I	py	-Q- lo m m — -o' — m m^m_m co co co co	co -a1____co -o1	ct co	-c
I	u_ ~	cd
I	® ! UOtJBJWAa .	_	......	"'t
® 1 jo	’diuaj. ci	— ad o' ci cd o' o' cd — -*	-o< ci ci tri cd id	cd ci	’d ©	od cd	o
El	co	Ti<-T<i.om-o'-^<-o,>.om-o'	m co ~c co CT co	coco	co -o’	c; co	’f
c .---------------------------------------------------------- JO-
§	. ul. A I	......................... . .	....'*.
2 S	^. aJHJ.a'uaj,|	cd —CTcdcdcicimcdcd	tri trd cd © ci cd	-d CT	tried	ci tri	cd
h	® |	______|	-o< m m uo-?-o1uo m-a1	m co-q*-fe CT co	tofe_______co -o*	ct co	-fe
z g„8 -a	aa . .w a“» .	& . .	.^'
M S = hd £ oded^^^’ eded^	^cd^cdcdcd
®	f=< 5 ?	.	...............................................................
R	_<	— — ct ct — — © CT © CT	CO — lO © CO CT_IIP t— CO -fe t,O I—	ct ©_
spno[3 jo j.uiy	©	©m©©©©©©©©	-fe © © © m ©	© m © o © co	©©	|
—
® -UOIJW.dBA^	.	.	.	................................................ t-:
o	io	-daw r	tried	—	cimirf-^t^-dadcicdt—	co - oocsCQm- ocigomtc oo	co
_	jo	utudj,	-^-^<co-rc'icococococococoM<-^coco	-o<
.	£	I	....................... GJ
—	KLq co	od	ui	o'od pi — ad c-i	o	ad	—	®«5®is?sj^£:S£2!222i2S£i22	—
p-	Ico -r<	co	—c	m__L.o_LO —f to	to	to	to_m_-c_co -r co co co co — co —- —r — co co
® g	P2f4 • MM	•. • •	u?u/
t? fegg'S-S	ai M	(z; co cd «5 co co P ?
u	r-j	—J cd cd — co' —i —	•■* cd cd iriuo'cdcimcd'^cdudidcd'^cdcd _
spnoojoj.mv]	o = o © © o oo c ci ©©©©©©©©cmcooooo©	|
- - --------------------------------------------------- •
t •uonu.dBAq	...............................
® JO -duiax ^^gJgj^^m^coco^m^^^moiMcl ct ys ” co co co co w CT CT co
g --------:-----------------------	-	.......... „
. g wnj.daxax ooUj^m'^toatCocitdtdK® weitsiSSSKSSS	—	2
53 |	>/* re -H4	’H’ UO ITS -rF co 'rr -J< ifi -7*	lGC^ CCfQGstwTG>QCOCQCO^CO^(NC>Q 
®_ g pt .KM . -M MK^ t-*. . • • .	si ^" < >.'
5	=1 g°s	^^czicci^^^aiccaicQ ai	k; i^i «2 !z; co !z; co	cd r* co
co’	—‘ — cd —‘ ci — cd — —-* cd ct ©-c co co co — co t-co —■-r — — ct —______
spnojojo j.tu y	©	© ° © ©t^cT© © oo oo ©© ° ^ —	ic'‘T*'O2 — 2°° —	5*00500
I— — co-o’co ct co-e m co — mCT ©-*	_ m oo m ©	© c? m . CT co
-UIO.I'Bfl d^^'^lloooo'ciooci©©©©©©©©©©©©©©©©©©©©©©	©
R	i CO CO CO CO CO CO COCOCOCOCOCOCOCOCOCTCOCOCOCOCTCOCTCO CT CT CT CT © ©_I CO
5------------tTP	pc	<—.	o	©	m	© m	m
5	2	ct	—	-3<	©	m	© ©	ct
® -upH jo 'UI —	*2	"fej	<7?	2	®i	2
-------^7, - ®Tcd rm~© i- ^ © © - ct g 2 2 2 iZ _ 2 ?, ? a] q, ?,	=; 3 _ | _
				

